# Mental distress in workers at two metallurgical companies in the state of São Paulo, Brazil

**DOI:** 10.47626/1679-4435-2021-705

**Published:** 2021-12-30

**Authors:** Phelipe Monteiro Felício, Arthur Arantes Cunha, João Silvestre Silva-Junior

**Affiliations:** 1 Curso de Especialização em Medicina do Trabalho, Faculdade de Ciências Médicas da Santa Casa de São Paulo, São Paulo, SP, Brazil.; 2 Curso de Graduação em Medicina, Departamento de Ciências Biológicas e da Saúde, Universidade Federal do Amapá, Macapá, AP, Brazil.; 3 Programa de Bolsas de Iniciação Científica, Departamento de Pesquisa, Universidade Federal do Amapá, Macapá, AP, Brazil.; 4 Departamento de Medicina, Centro Universitário São Camilo, São Paulo, SP, Brazil.

**Keywords:** mental health, occupational health, ergonomics, mental disorders, saúde mental, saúde do trabalhador, ergonomia, transtornos mentais

## Abstract

This study aimed to map the prevalence of mental distress among employees at two metallurgical companies and to analyze differences according to the sex of workers and the employing company. An analytical cross-sectional study was conducted using the 20-item Self-Reporting Questionnaire to map the prevalence of mental distress. A total of 439 workers participated in the study. The overall prevalence was 10.0%, being 4 times higher in women (32.3%) than in men (8.3%) (p < 0.001). There was no significant difference between the prevalence rates of mental distress according to employing company (p = 0.271) or sector (p = 0.239). The most frequent complaint was ‘nervousness, tension, and worry’ (48.7%). These results indicate the need for management of workers’ mental health, especially among women.

## Resumo

Objetivou-se mapear a prevalência de sofrimento mental entre empregados de duas empresas metalúrgicas e analisar diferenças conforme o sexo dos trabalhadores e a empresa de vínculo. Tratou-se de um estudo transversal analítico que utilizou o questionário Self-Reporting Questionnaire de 20 perguntas para mapear a prevalência de sofrimento mental. Participaram da pesquisa 439 trabalhadores. A prevalência geral foi 10,0%, sendo cerca de quatro vezes maior no sexo feminino (32,3%) que no masculino (8,3%) (p < 0,001). Não houve diferença entre as prevalências de sofrimento mental segundo empresa de vínculo (p = 0,271) ou setor (p = 0,239). A queixa mais frequente foi “nervosismo, tensão e preocupação” (48,7%). Esses resultados indicam a necessidade de gerenciamento da saúde mental dos trabalhadores, especialmente das mulheres.

## INTRODUCTION

Mental and behavioral disorders are chronic clinical conditions that affect a large proportion of people around the world and generate substantial socioeconomic costs. The impact of psycho-emotional disorders on the working ability of Brazilian workers was the third leading cause of paid temporary work disability benefits between 2012 and 2016 in Brazil.^1^ Depression and anxiety, common disorders resulting from mental distress, were the main reasons for workers receiving these benefits. Mental disorders lead to longer sick-leave periods than other morbidities and have a greater budgetary impact in terms of expenditure for the public national social security system.^1^

Metallurgical companies directly employed an average of 234,000 workers between 2013 and 2015 in Brazil. Their importance for the labor market extends into the production chain of other segments, such as the automotive industry, construction, and capital goods. This economic activity has a direct impact on national wealth, accounting for 5.3% of the industry’s gross domestic product and 8.4% of Brazilian exports according to 2015 data.^2^ Faced with this scenario, it is important to consider the negative socioeconomic impact of mental illness on metallurgical workers in terms of direct costs, such as health expenses, and indirect costs related to decreased productivity, presenteeism and absenteeism due to illness. Therefore, mapping subclinical cases may be useful in the planning of actions aimed at promoting health and preventing mental disorders.

In view of the foregoing, the present study aimed to map the prevalence of mental distress among employees at two metallurgical companies in the state of São Paulo, Brazil, and to analyze differences in its frequency according to the sex of workers and the employing company.

## METHODS

We conducted an analytical cross-sectional study of workers from two metallurgical companies located in the city of Osasco, metropolitan area of São Paulo, Brazil, in 2018. A total of 439 workers participated in the study: 147 from company A and 292 from company B. Company A produces steel wire for several areas, such as the construction industry, and company B produces steel cables for the oil and mining industry.

At the time of the periodic health examination at each company, all workers were asked to complete the Brazilian Portuguese version of the 20-item Self-Reporting Questionnaire (SRQ-20).^3^ The SRQ-20 is a self-administered questionnaire with a dichotomous scale (yes/no) for each of the questions. It was designed to screen for mental disorders, but it does not provide a specific diagnosis, and its use is recommended for studies at the population level.^3^ Participants were considered to be experiencing mental distress if they provided 7 or more positive answers, regardless of the participant’s sex.

The chi-square test was used to analyze whether there were differences between the sexes and between the employing companies in cases of mental distress. Differences in the answers to the SRQ-20 questions were also analyzed according to sex. A p-value < 0.05 was considered significant. Total Cronbach’s alpha was calculated to analyze the internal consistency of the SRQ-20, with further removal of each item, and interpreted as follows: very weak (0 to 0.20), weak (0.21 to 0.40), fair (0.41 to 0.60), good (0.61 to 0.80), very good (0.81 to 0.92), and excellent (0.93 to 1.00). Statistical analysis was performed using Microsoft Excel for Office 365 MSO and RStudio, version 1.2.5033.

The study was approved by the Research Ethics Committee of the Irmandade da Santa Casa de Misericórdia de São Paulo (approval number 2.573.262/2018) and registered at Plataforma Brasil (CAEE 79777817.1.0000.5479). Written informed consent was obtained from each study participant.

## RESULTS

Of the 439 workers who participated in the study, 292 (66.5%) were from company A and 147 (33.5%) from company B, with a similar percentage distribution according to the sector of employment: production line (77.8%-78.9%) and administrative department (22.2%-21.1%). The overall prevalence of mental distress was 10.0%, with a rate of 8.9% (26/292) in company A and 12.2% (18/147) in company B. There was no statistically significant difference according to employing company (p = 0.271) or sector (p = 0.239). Among the 408 male participants, the prevalence of mental distress was 8.3% (34/408). Among the 31 female participants, the prevalence was 32.3% (10/31), with a statistically significant difference in the results between men and women (p < 0.001).

The most frequent complaint was “nervousness, tension, and worry” (Q6 - 48.7%), followed by “poor sleep” (Q3 - 34.4%). The least reported question was the one about thoughts of ending life (Q17 - 0.5%). Most questions had a higher positive frequency among women. The items with no statistically significant difference between the sexes were as follows: poor sleep (Q3), unhappiness (Q9), frequent crying (Q10), inability to play a useful part in life (Q14), worthlessness (Q16), and thoughts of ending life (Q17) (Table 1).

**Table 1:** Distribution of participants according to sex and positive answers to each of the 20 questions of the Self-Reporting Questionnaire (SRQ-20), 2018, Osasco, São Paulo, Brazil (n = 439)

		Yes n (%)	M n (%)	F n (%)	p-value
Q1	Do you often have headaches?	76 (17.3)	63 (15.4)	13 (41.9)	< 0.001
Q2	Is your appetite poor?	47 (10.7)	40 (9.8)	7 (22.6)	0.027
Q3	Do you sleep badly?	151 (34.4)	136 (33.3)	15 (48.4)	0.090
Q4	Are you easily frightened?	74 (16.9)	62 (15.2)	12 (38.7)	< 0.001
Q5	Do your hands shake?	45 (10.2)	37 (9.1)	8 (25.8)	0.003
Q6	Do you feel nervous, tense, or worried?	214 (48.7)	190 (46.6)	24 (77.4)	< 0.001
Q7	Is your digestion poor?	76 (17.3)	62 (15.2)	14 (45.2)	< 0.001
Q8	Do you have trouble thinking clearly?	62 (14.1)	52 (12.7)	10 (32.3)	0.003
Q9	Do you feel unhappy?	95 (21.6)	84 (20.6)	11 (35.5)	0.052
Q10	Do you cry more than usual?	24 (5.5)	20 (4.9)	4 (12.9)	0.080
Q11	Do you find it difficult to enjoy your daily activities?	67 (15.3)	53 (13.0)	14 (45.2)	< 0.001
Q12	Do you find it difficult to make decisions?	72 (16.4)	60 (14.7)	12 (38.7)	< 0.001
Q13	Is your daily work suffering? Extremely painful? Do you find it difficult to get your work done?	35 (8.0)	29 (7.1)	6 (19.4)	0.015
Q14	Are you unable to play a useful part in life?	20 (4.6)	19 (4.7)	1 (3.2)	1.000
Q15	Have you lost interest in things?	46 (10.5)	36 (8.8)	10 (32.3)	< 0.001
Q16	Do you feel that you are a worthless person?	18 (4.1)	17 (4.2)	1 (3.2)	1.000
Q17	Has the thought of ending your life been on your mind?	2 (0.5)	2 (0.5)	0 (0.0)	1.000
Q18	Do you feel tired all the time?	71 (16.2)	59 (14.5)	12 (38.7)	< 0.001
Q19	Do you have uncomfortable feelings in your stomach?	86 (19.6)	71 (17.4)	15 (48.4)	< 0.001
Q20	Are you easily tired?	96 (21.9)	81 (19.9)	15 (48.4)	< 0.001

F = female; M = male.

As for the internal reliability of the questionnaire, the Cronbach’s alpha value was 0.8024, ranging from 0.7599 to 0.7866 for item-by-item reliability.

## DISCUSSION

The overall prevalence of mental distress in our sample was lower than that found by Farias & Araújo^4^ in a sample of 1311 workers, in which a rate of 25.2% was observed. The lower overall prevalence found in the present study may be due to the characteristics of the sample, since the absolute majority of participants were men, whereas in the study by Farias et al., men represented half of the sample.^4^ However, both studies agree in that there was a statistically significant difference between the sexes, with a higher rate of psycho-emotional stress among women.^4^

Aspects related to the female sex, such as the burden of a double shift (waged and domestic labor) and their ease in reporting signs/symptoms, may be a determinant of a higher rate of psychological distress. Considering that paid disability benefits resulting from mental disorders are more common among women and often lead to longer sick-leave periods, which, in turn, leads to a greater economic impact on the social security system,^1^ it is necessary to consider women a population at risk for psycho-emotional disorders. Therefore, strategies to promote mental health and prevent disorders at the workplace should implement specific actions for women.

In the study by Guirardo & Pereira^5^ of metallurgical workers from a company in the countryside of São Paulo, also using the SRQ-20 to assess the mental health of workers, the questions about “nervousness, tension, and worry” and about “poor sleep” were also the most commonly reported ones by the participants. This may be related to the characteristics of the tasks performed by many workers in the metallurgical industry, which involve continuous attention, tension due to the risk of accidents, and rotating schedules, among others. Such characteristics of this economic activity may explain the lack of statistical significance regarding the frequency of mental distress among workers when comparing two companies within the same industry located in the same region of São Paulo State.

The data from the present study showed good internal reliability of the instrument used to assess mental distress. The World Health Organization (WHO) recommends the use of the SRQ-20 for screening in the assessment of mental health because it is an easy-to-understand, quick-to-administer, low-cost instrument whose international standardization allows for comparisons. Therefore, it is feasible to indicate the use of this questionnaire in occupational health services in order to expand and improve the assessment of workers’ health.

## CONCLUSIONS

This study found that 1 in every 10 workers of two metallurgical companies in the state of São Paulo experienced mental distress as assessed by a standardized questionnaire. This condition was associated with the female sex, but there was no difference in its frequency according to the worker’s employing company or the characteristics of the sector of employment. The specific complaints were similar to those reported in a previous Brazilian study of workers from the same economic branch.

In view of the potential damage, with regard to reduced productivity and sick leave in an important industrial sector that generates jobs and is economically relevant, it is necessary to encourage the management of workers’ mental health in Brazilian metallurgical companies. Preventive measures are needed at the primary level, such as those related to the management of stressors at the workplace and to the support required to cope with everyday problems, at the secondary level, such as early-diagnosis strategies to refer workers to specialized care, and at the tertiary level, such as monitoring of whether health care is appropriate to the clinical condition and psychosocial rehabilitation. These interventions can help to achieve positive results in preventing mental distress and promoting the overall health of individuals.
